# Chemotherapy Release From Bortezomib-Impregnated Polymethylmethacrylate-Coated Intramedullary Nails: A Novel In Vitro Study for a Local Chemotherapy Delivery Device

**DOI:** 10.7759/cureus.64181

**Published:** 2024-07-09

**Authors:** Emily Ren, Weiping Ren, Angela C Collins, Andrew Robinson, Rahul Vaidya

**Affiliations:** 1 Department of Orthopaedic Surgery, Wayne State University Detroit Medical Center, Detroit, USA; 2 Department of Biomedical Engineering, Wayne State University, Detroit, USA; 3 Department of Orthopaedic Surgery, The Ohio State University Wexner Medical Center, Columbus, USA

**Keywords:** pmma, localized chemotherapy, elution, orthopedic tumors, bortezomib, cement, multiple myeloma

## Abstract

Bortezomib (BAN) is a proteasome inhibitor approved for the treatment of multiple myeloma and lymphoma. Despite its efficacy in various tumor models, systemic administration can result in toxicity to healthy organs. The purpose of this study is to evaluate the elution profile of BAN from PMMA cement for the local treatment of orthopedic tumors. BAN solution (5 mg; 2 mg/mL) was mixed with Simplex cement (40 g, Stryker), followed by injection of cement into an antibiotic cement nail mold (13 mm) to coat a 10 mm titanium femoral nail (DePuy Synthes). Once the cement polymerized, the nail was cut into 2 cm segments for the BAN elution study. There is a sustained release of BAN for up to 28 days. The overall concentration of BAN released at each time point was between 74 and 263 ng/ml, which is compatible with the peak blood concentration of a single intravenous BAN injection. This study demonstrates the feasibility of using PMMA bone cement as a local BAN delivery tool, essential for future studies and treatment targeting multiple myeloma cells.

## Introduction

Many efforts have been made to develop controlled local drug delivery devices with less or no systemic toxicity. The search for local drug delivery carriers is necessary as most systemic anticancer drugs are considerably toxic to normal cells as well, preventing the use of high dosages [1]. Bortezomib (BAN) (Fresenius Kabi Pharmaceuticals, IL, USA) is a proteasome inhibitor approved and used in the United States and Europe for the treatment of multiple myeloma and mantle cell lymphoma [2]. BAN targets the 20S proteasome and the ubiquitin-proteasome system (UPS) in cancer. BAN exerts its anti-cancer effects through various mechanisms, including generation of reactive oxygen species (ROS), suppression of the unfolded protein response, accumulation of ubiquitinated misfolded proteins that lead to the endoplasmic reticulum stress, inhibition of the cellular NFκB survival pathway, and stabilization of tumor suppressor proteins: cyclins, p27, and p53 [2,3]. 

The UPS, a complex and dynamic system, has a critical role in the functioning of both normal and cancer cells. The system is responsible for the majority of degraded proteins within the cell. Cancer cells take advantage of the UPS to maintain cell growth and resist apoptosis. Therefore, proteasome inhibitors, such as BAN, focus on disrupting the UPS. By disrupting the UPS, cells experience modulation of signaling pathways, cellular activities, apoptosis, and autophagy. The UPS functions as a post-translational modification, affecting the structure of proteins through the addition of ubiquitin (Ub) moieties [2-4]. 

At the core of the UPS is the 26S proteasome, which consists of a 20S catalytic core and two 19S regulatory caps. The 20S catalytic core, which is the proteasome target of BAN, is responsible for enzymatically degrading ubiquitinated proteins. The 19S regulatory caps control the removal and recycling of ubiquitin moieties linked to proteins and bring targeted proteins to the end of their fate in the 20S proteasome. Ubiquitin must be recycled for future degradation of intracellular proteins. Ubiquitin has a plethora of complex branching patterns that significantly influence the behavior of target proteins, offering a wide array of potential targets for future UPS-targeted drugs, separate from that of BAN and other proteasome inhibitors [3-5]. 

Protein ubiquitination, a multi-step ATP-dependent process, involves Ub-activating (E1), Ub-conjugating (E2), and Ub-ligating enzymes (E3), individual components of the UPS. The degradation of the proteins occurs at E3, whereas E1 and E2 function to tag the protein for breakdown. Protein ubiquitination allows for the tagging of proteins to be degraded by 26S proteasome. This enzymatic cascade allows for specific modulation of protein degradation, influencing cellular activities and functions through protein binding specificity and differential expression of UPS components [3-5]. 

The UPS regulates crucial cellular pathways, including cell-cycle progression, apoptosis, and the NFκB survival pathway. Cancer cells exploit the UPS, which enables cells to continue the accelerated growth and resist the normal cell apoptosis cycle. It is for this reason that the UPS is a viable target for therapeutic intervention by proteasome inhibitors. BAN, through proteasome inhibition, induces cell-cycle arrest, apoptotic cell death, and modulation of various cellular pathways, offering a promising strategy for therapeutic effects against multiple myeloma [2,3,5].

In vitro, multiple myeloma cells don’t have the same sensitivity to BAN-induced proteasome inhibition and apoptosis compared to normal cells because it inhibits the NFκB survival pathway, which is more utilized by myeloma cells. During normal cell functioning, the NFκB pathway is inhibited from increasing transcription and activation of growth-related genes by IκB. Myeloma cells depend on the NFκB survival pathway to allow for their continual and accelerated division, thus requiring greater protein turnover by proteasomes. BAN’s ability to disrupt these processes, coupled with its effects on pro-apoptotic and anti-apoptotic genes, highlights its potential to induce selective cancer cell death. Despite increased selectivity to MM cells, BAN along with most systemic anticancer drugs is still considerably toxic to normal cells [3,6-8]. 

The main issues associated with BAN, as with most proteasome inhibitors and anticancer drugs, include toxicities, non-targeted symptoms, potential resistance to the drug or other chemotherapeutic drugs, and multidrug resistance. Resistance to BAN, or anticancer drugs, can be inherent or acquired depending on the individual. Some of the common non-targeted symptoms associated with the toxicities of BAN are gastrointestinal side effects, myelosuppression, and neurotoxicity. The latter, often peripheral sensory neuropathy, has been addressed through subcutaneous administration, preserving the anti-cancer effects of the drug while mitigating neurotoxicity. It is for this reason that most institutions have adopted subcutaneous administration of BAN. The exploration of BAN’s mechanisms of action and its impact on the UPS provides a comprehensive understanding of its role in cancer therapy, especially multiple myeloma in the context of this paper [3,9,10]. 

Multiple myeloma is characterized by an abnormality of bone marrow plasma cells that proliferate. It is diagnosed as follows: (i) clonal bone marrow plasma cells ≥10% or biopsy-proven bony or extramedullary plasmacytoma plus and (ii) end-organ damage caused by the plasma cell disorder resulting in hypercalcemia, renal insufficiency, anemia, or bone lesions [11]. 

Plasma cell accumulation in the bone marrow leads to osteolytic bone lesions by interfering with bone remodeling by uncoupling osteoblastic bone formation and osteoclastic bone resorption. The osteoclasts dominate, resulting in osteolytic lesions [12]. At the time of diagnosis, 80% of patients with multiple myeloma have osteolytic lesions. Osteolytic lesions may cause patients to have significant bone pain and a potential risk of life-threatening hypercalcemia [12]. Due to the nature of multiple myeloma, as it relates to low bone density and lesions, there is an increased risk of skeletal complications with 40% of multiple myeloma patients experiencing pathological fractures throughout the course of the disease. Pathological fractures are associated with an increased mortality risk, increasing odds between 23 and 32%. However, in contrast, the detection and treatment of impending pathological fractures is associated with decreased morbidity and mortality [11,13]. 

Therefore, orthopedic surgeons determine prior to the presentation of pathological fractures whether patients are at risk in order to identify patients who may benefit from prophylactic fixation. Surgeons evaluate the risk using Mirels’ criteria. There are four components when accessing a patient with lesions: site of lesion, size of lesion, nature of lesion, and pain. Each component is associated with a score from one to three, where three is the most severe risk of pathological fractures. Patients with a cumulative score of less than or equal to eight are treated non-operatively while patients with a cumulative score greater than or equal to nine are treated through prophylactic stabilization [14,15].

The most common structural components of the skeleton targeted by multiple myeloma include the long bones (femur, tibia, humerus) and the spine [11]. The treatment is based on obtaining a definitive diagnosis [15], then supporting these structures by intramedullary nailing of the long bones [16-19], and in the case of the spine supporting the vertebra with either bone cement or structural spacers with or without pedicle screw instrumentation [20]. 

Although BAN is an effective drug, it has many side effects on healthy organs [1,2,6]. Also, its bioavailability in the body is limited due to its low water solubility and instability in conventional chemotherapy [2]. Delivering BAN locally could theoretically allow for much higher local concentrations without the systemic effects [2]. Currently, subcutaneous injection guidelines for BAN suggest a maximum subcutaneous injection of 2 mL, where the concentration for the drug is 2.5 mg/mL. BAN can also be delivered through intravenous injection with a concentration of 1 mg/mL. Our institution mainly injects BAN subcutaneously as do most institutions [1]. 

Kruppke et al. have already demonstrated the release of BAN from calcium phosphate-containing silica/collagen xerogels [2]. Polymethylmethacrylate (PMMA) is widely used in the field of orthopedic and trauma surgery. It is the principal component for many bone cements across the market and is employed for implant fixation, void filling, and bone reconstruction. It is a relatively cheap and FDA-approved material for use in the human body. Most PMMA cements are offered as a two-component system, a liquid monomer and PMMA powder, for clinical ease. As these two components are mixed, the mixture undergoes an exothermic reaction as it transitions from a pliable state to a solid state when curing is finished. Due to the plasticity of PMMA cement, it can be shaped to the needs of the surgical procedure [21]. Furthermore, PMMA bone cements serve as carriers for antibiotics, especially after infections in total hip arthroplasty, total knee arthroplasty, and long bone infections. Antibiotic-impregnated PMMA can be used to deliver antibiotics locally in addition to the administration of intravenous or oral antibiotics to infected bone environments. The antibiotics mixed within the cement elute over a period of time, some for several months. This dual functionality combines stable fixation with anti-sepsis principles, displaying the significance of antibiotic-loaded bone cement in arthroplasty and fracture surgery. PMMA is a potent tool for managing complex musculoskeletal conditions as it has structural functionality and a controlled release of medication over time, allowing for potentially higher doses compared to systemic administration [2]. The concept of using PMMA as a delivery device for chemotherapeutic agents is new and has not been tested with BAN. Bone tumor patients with metastatic tumors, primary sarcomas, and multiple myeloma often require structural support for pathologic fractures and impending pathologic fractures in long bones with intramedullary nails where the tumor has eroded the bone. A coating device (ABC (Antibiotic Cement) Nail Mold (Bonesetter Holdings Ann Arbor USA) was developed for coating intramedullary nails with PMMA and antibiotics in cases of long bone infections or osteomyelitis [22,23]. It is sized 11, 13 mm inner core, allows no touch technique especially important for handling chemotherapeutic agents and simply peels off when the PMMA mixed with antibiotics has set. 

The purpose of this study is to (i) evaluate if we can make BAN-impregnated PMMA-coated intramedullary nails that have a consistent drug makeup and (ii) establish if BAN elutes from such a device in a similar manner to antibiotics-coated nails.

## Materials and methods

Nail and nail mold

We used a 9 mm femoral titanium cannulated antegrade retrograde nail (DePuy Synthes, Paoli, Pennsylvania) and Stryker Simplex Cement (Stryker, Mahwah, NJ USA) for PMMA cement coating which is 41 gm of sterile powder (contains 6 gm of Polymethyl methacrylate, 30 gm of methyl methacrylate-styrene-copolymer, 4gm of barium sulfate) and 20 mL of sterile liquid (contains 19.5 ml of methyl methacrylate, 0.5 ml of N, N-dimethyl-para-toluidine and 75ppm Hydroquinone). A 13 mm ABC (Antibiotic Cement) Nail Mold (Bonesetter Holdings, Ann Arbor, USA) was used for the cement coating with a Stryker cement mixing setup and cement gun (Stryker, Mahwah, NJ USA). 

Ready-to-use BAN solution (final 2.5 mg/ml in saline) was provided by our pharmacy in the exact mixture that is used for subcutaneous administration.

Femoral titanium cannulated nail with PMMA cement coating and BAN doping

To create a proper mixture, we attempted several methods of preparing the PMMA cement mixture. Antibiotics are usually in a powder crystalline form and can be mixed with the powder polymer component of bone cement. They have a volume of approximately 10 cc so the material is easy to mix. However, the BAN solution comes in a low-volume liquid of 1 or 2 cc and this is very difficult to mix evenly with the 40 gm of powder cement. Thus the BAN solution was added to the liquid monomer, rather than the dry powder, and mixed thoroughly in a syringe. McLaren et al. found that adding antibiotics to the monomer or the PMMA powder has no difference in the cumulative release of the antibiotics [24]. The dry powder cement mix (40 g-one package) and its monomer (20ml) mixed with 2 ml of BAN solution (total 5 mg) were mixed in a cement mixer (Revolution Cement Mixing System; Stryker, Mahwah, NJ USA), ensuring no clumps were left and the handling of the chemotherapeutic agent. We didn’t use vacuum mixing as that reduces porosity which is desirable for cementing arthroplasty components but increased porosity is better for antibiotic elution from PMMA cement and we felt the same would apply for BAN [25,26]. The cement mixture was emptied into the cement canister, attached to the Stryker cement gun. The 13mm ABC nail mold was attached to the Stryker canister and then the cement mixture was injected into the ABC nail mold with the gun/canister apparatus in a very liquid form. We then inserted the 9mm Synthes IM femoral nail into the mold from the other end. This was inserted up to the proximal locking holes. When the cement hardening reaction was finished, (we checked for cement hardening by removing the cement gun and checking the end of the cannister) approximately 10 minutes. The ABC mold was then peeled off with its handles [27]. The cement-coated nail was then cut with a band saw into 2 cm sections. We took samples from the distal, middle, and proximal parts of the nail to test if there was even distribution of the drug through the coating procedure. We used three samples that were equal in weight to ensure the gross cement coating was similar to the previously described [25,26]. 

BAN elution protocol

Cement segments were placed into capped 15 mL Falcon polypropylene tubes (Becton Dickinson, Franklin Lakes, New Jersey) containing 6 mL sterile phosphate-buffered saline at room temperature. The segments were removed and replaced in fresh phosphate-buffered saline at 15 and 30 minutes, and at one hour, four hours, eight hours, one day, two days, three days, seven days, 14 days, and 28 days, respectively. These eluted samples were stored at -20°C until the end of the four-week sampling period. The concentration of BAN released was measured by the characteristic absorption maximum at a wavelength of 270 nm (Synergy HT, BioTek Instruments, and Winooski, VT), as described by Chaudhary et al. [28].

## Results

Handling and cutting

**Figure 1:**
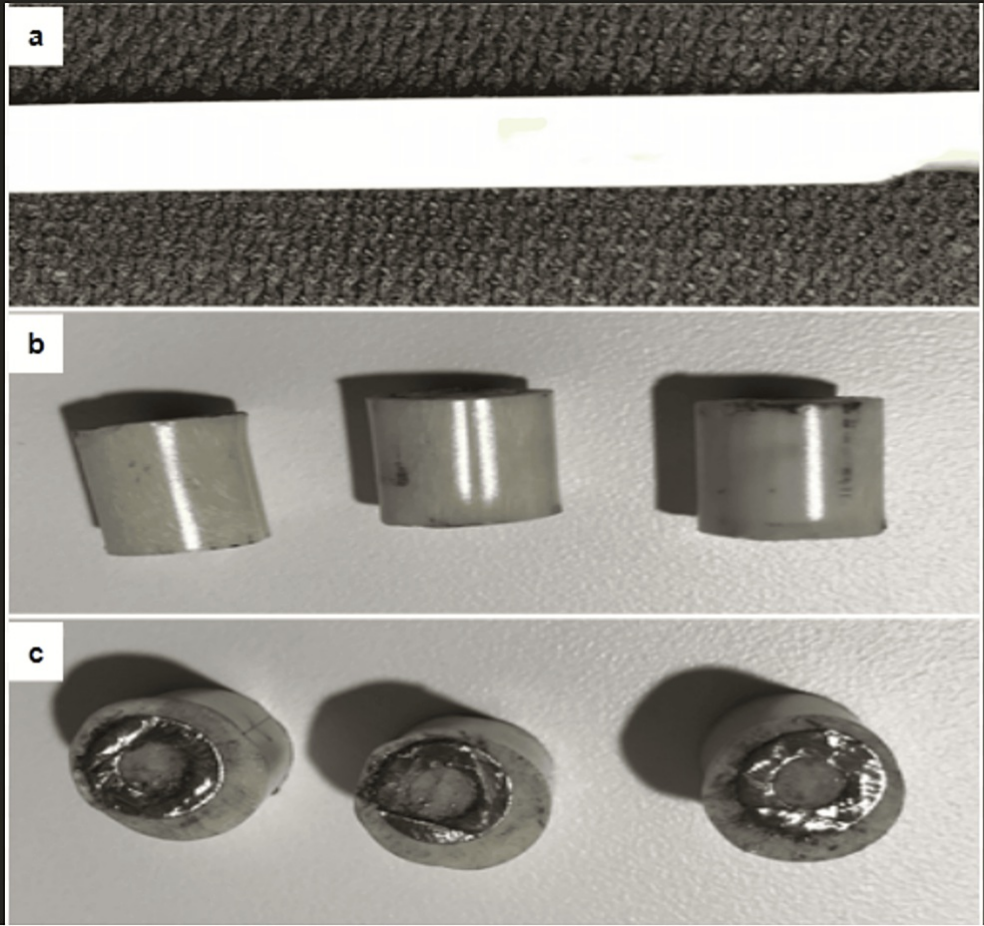
Morphology of cannulated nail with polymethylmethacrylate cement and bortezomib doping (a) Nail with cement coating right after removal of the chest tube. (b) Surface of polymethylmethacrylate cement coating segment after cutting. (c) Cross section of cement segment after cutting showing the thickness of cement coating.

The addition of BAN at the current amount, 2 ml, has little impact on cement coating, setting time, and ABC nail mold removal after setting (no cement residue was observed on the removed nail mold). In addition, the cutting of a 10 mm cannulated nail with BAN-doped PMMA cement coating into 2 cm segments is feasible and without any difficulties. The PMMA coated the outside and the inside of the intramedullary nail and was measured to be a consistent 13 mm in diameter even though the coating was not consistent coating around the nail. There were no visible cracks, fractures, or delamination on the surface of the around 2 mm-thick cement coating from the canulated nail during band saw cutting (Figure 1).

BAN elution

**Figure 2:**
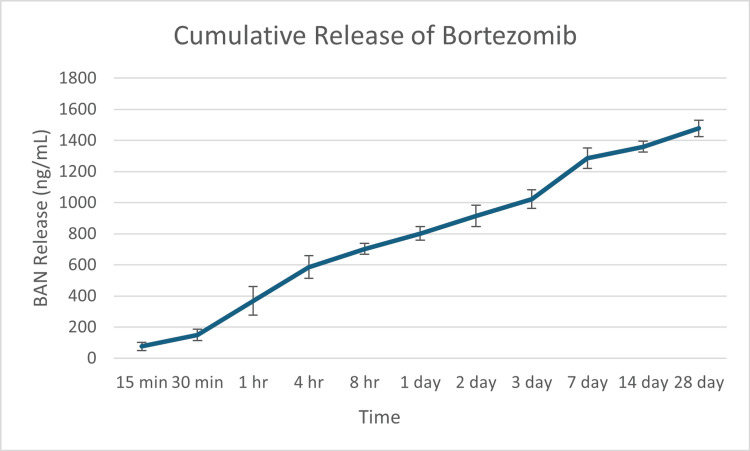
Bortezomib release from polymethylmethacrylate cement coating Cumulative bortezomib release from polymethylmethacrylate cement coating over the course of 28 days (n = 3) BAN: Bortezomib

We embedded 5 mg of BAN in 40 grams of Simplex P cement for nail coating. The elution of BAN from PMMA cement coating for up to 28 days was studied and the results are shown in Figure 2 and Table 1. In contrast to the burst release of vancomycin and tobramycin from PMMA cement within the first 48 hours, as described before [26], a sustained, near zero-order release of BAN was observed starting from 30 minutes and up to 28 days. The net release of BAN at each time point is shown in Table 1. The overall concentration of net BAN released at each time point was between 74 and 263 ng/ml, which is compatible with the peak blood concentration of a single intravenous BAN injection and sufficient for inducing cell toxicity of multiple myeloma cells [29]. Figure 2 shows the cumulative elution of BAN over the 28-day course, 1359 ng/ml.

**Table 1:** Net elution of bortezomib at given times (ng/ml) The table shows the net release of bortezomib at given times. It shows that the concentration of bortezomib released from polymethylmethacrylate cement coating over the course of 28 days was stable and between 74 and 263 ng/ml, which is compatible with the peak blood concentration of a single intravenous bortezomib injection.

15 min	30 min	1 hr	4 hr	8 hr	1 day	2 day	3 day	7 day	14 day	28 day
76±26	74±37	218±92	218±72	116±35	100±43	113±70	107±60	263±65	74±35	119±53

In addition, there was a consistent and similar release from the three samples at each time period owing to the consistent mixture that coated the nail.

## Discussion

We performed this study to assess if we could add BAN to PMMA and get a timely elution of the drug to the local environment. In addition, we did this study using a cement-coated intramedullary nail device because this is exactly what we would use to fix or support pathologic fractures or impending fractures of long bones [15-19]. The use of antibiotic cement-coated intramedullary nails is commonplace in orthopedic surgery for fracture-related infections of long bones (femur, tibia, humerus) [21-23] to deliver antibiotics locally to fight infection. So why not chemotherapeutic drugs? 

Once a femur, tibia, or humerus is stabilized with an intramedullary nail for a pathologic fracture or impending pathologic fracture, the bone is further treated with radiation to address the possibility of seeding the tumor through the entire length of the bone. This is usually done once the skin has healed after surgery 2 to 3 weeks post-op [16-19]. Having the ability to deliver a chemotherapeutic agent directly to the tumor itself and the local environment within the medullary canal is potentially a powerful tool in the treatment of metastatic or primary tumors of the long bones. 

There have been prior studies on the release of other chemotherapeutic drugs such as methotrexate from local carriers including polymeric dicalcium phosphate dehydrate cement [30] and PMMA as long ago as 1999 [23]. Decker et al. [23] described the use of bone cement (PMMA) as a delivery device containing methotrexate dissolved in N-methyl-pyrrolidone which released the drug in a suitable and effective way. It was felt to be of value in the treatment of bone tumors. Kruppke et al. [2] have demonstrated the release of BAN from calcium phosphate-containing silica/collagen xerogels. We have shown the same from polymethylmethacrylate (PMMA, Stryker Simplex Cement (Stryker, Mahwah, NJ USA)) which is an FDA-approved product for use in orthopedic surgery. PMMA is available as a sterile product and is commonly available worldwide for about 60 dollars a package [21-26]. Thus a low-cost alternative and available drug delivery substrate for this chemotherapeutic drug. 

We were unable to find any prior studies that have examined the release of BAN from PMMA and no prior study on any chemotherapeutic agent coated onto intramedullary nails as a local drug delivery device. 

The dose of BAN that we chose for the experiment was exactly what is used as a single dose subcutaneously for a 70 kg person [1]. The reason for this was simple and that is if the whole dose was somehow completely delivered into the bloodstream it was no more than what is routinely given and should not have any more toxicity than what is the standard. The addition of BAN at the current amount has little impact on cement coating, setting time, and nail mold removal after setting (No cement residue was observed on the removed ABC nail mold). This shows the feasibility of BAN use with PMMA as there are antibiotics additives, such as rifampin, that despite being in their crystalline state inhibit the curing process of PMMA [21]. 

In addition, the cutting of a 10 mm cannulated nail with BAN-doped PMMA cement coating into 2 cm segments is possible. Though it is difficult to make consistent cement thickness coating on the nails, there were no visible cracks, fractures, or delamination on the surface of the around 2 mm-thick cement coating from the canulated nail during band saw cutting (Figure 1). We prepared the PMMA cement at room temperature, which has been shown to only affect the settling time [31]. Thus, it has no effect on the outcome of the 2 cm segments. In addition, we prepared the mixture in an open bowl as it increases the porosity of the cement compared to when prepared with vacuum, allowing better elution of the BAN [32]. The consistency of the mixture and preparation of the nail showed that elution was similar from the proximal middle and distal portions of the fabricated antibiotic nail throughout the collection periods which means we were able to mix the BAN solution evenly in the cement product. 

BAN is a modified dipeptidyl boronic acid and a proteasome inhibitor approved for use in the United States and Europe for the treatment of multiple myeloma and mantle cell lymphoma [2,5]. BAN is highly cytotoxic to all multiple myeloma cell lines in vitro [33], yet when used clinically, only 40% of myeloma patients are responsive to BAN when used as a single drug treatment [34]. This could be because we can’t get the same local concentrations or length of BAN exposure in clinical settings as compared to in vitro settings at the maximal tolerated dose (MTD) of the drug [34]. 

Most studies of BAN in cell culture have utilized continuous incubation for 24-48 hr. In the clinical setting, patients receive intravenous or subcutaneous bolus injections twice weekly [33]. When BAN is injected intravenously at the MTD, the blood plasma concentration peaks at 100-200 ng/mL five minutes after IV injection followed by a rapid decrease [34,35]. Subcutaneous injection results in a 10-fold lower maximal concentration, which is achieved 30 minutes after injection. The maximal concentration of the drug is maintained for 1-2 h so that total exposure to BAN (area under the drug concentration-time curve) is the same as after IV administration [36]. Thus, the efficacy of the agent does not depend on the administration route [37]. 

In the current study, we embedded 5 mg of BAN in 40 grams of Simplex P cement for nail coatings shown in Figure 2 and Table 1. A sustained, near zero-order, release of BAN was observed starting from 30 minutes and up to 28 days. This is similar to the release of Gentamicin which also experiences a continuous release kinetic from PMMA rather than a burst pattern where the majority of eluted medication occurs within the first 24 hours such as vancomycin [18]. The net release of BAN at each time point was between 74 and 263 ng/ml, which is compatible with the peak blood concentration of a single intravenous BAN injection and is sufficient for inducing cell toxicity of multiple myeloma cells [29]. 

Limitations of this study include the fact that this was an in vitro study and it is impossible to know the concentration achieved of the drug in the intramedullary canal of a human. We have done our best to use a model that has been used before when testing antibiotic elutions [19,20]. The dose we used was the same as used for a subcutaneous dose given to a 70 kg male but most drug elution studies show that only a percentage of the drug actually elutes from the PMMA. How much exactly does the drug elutes and the sustained intramedullary concentration is of course unknown. We used only three samples because we were interested in using the proximal middle and distal part of the nail with the weights of samples being the same. Other preparations with different dosages and multiple nails could certainly be tested to get better statistical analysis. We used only one nail but this was a feasibility study. The handling of chemo-doped cement has to be very careful, as even small doses of BAN are effective in cancer treatment and likely could affect normal tissue. The setup that was used allows everything to be mixed and poured in a no-touch technique until the nail has cured. This creates a constant elution between samples.

## Conclusions

This study demonstrates the feasibility and safety of using PMMA bone cement as a tool for local BAN delivery on an intramedullary nail. Our data confirmed that the time-dependent BAN released at the current dosage of BAN loading and nail coating was in the range that is compatible with the peak blood concentration (~200 ng/mL) used in a clinical setting by intravenous injection. These results are essential for future studies of in vitro cell culture of human multiple myeloma cells in the presence of eluents collected during drug release experiments. Local application of BAN has the benefit that higher concentrations of BAN can be achieved in the surroundings of the implanted material, leading to longer and sustained growth inhibition of local myeloma cells. Our results provide the basis for the local release of BAN from PMMA cement and represent a key step for successful treatment when nailing impending pathological and pathologic fractures.
